# OPM-MEG in multiple sclerosis: Proof of principle, and the effect of naturalistic posture

**DOI:** 10.1016/j.nicl.2025.103888

**Published:** 2025-09-26

**Authors:** Benjamin J. Sanders, Christopher G.S. Gilmartin, Lukas Rier, Lauren Gascoyne, Emily McCann, Jorge Cabrera, James Leggett, Niall Holmes, Ryan M. Hill, Elena Boto, Natalie Rhodes, Clarise Castleman, Aimee Hibbert, Daniel C. Ford, Holly Schofield, Cody Doyle, James Osborne, David Bobela, Vishal Shah, Karen J. Mullinger, Kathryn Radford, Matthew J. Brookes, Nikos Evangelou

**Affiliations:** a Sir Peter Mansfield Imaging Centre, School of Physics and Astronomy, University of Nottingham, University Park, Nottingham NG7 2RD, UK; b Division of Clinical Neurology, Queen’s Medical Centre, University of Nottingham, Nottingham NG7 2UH, UK; c Mental Health and Clinical Neurosciences Academic Unit, School of Medicine, University of Nottingham, UK; d Cerca Magnetics Limited, Units 7&8 Castlebridge Office Village, Kirtley Drive, Nottingham NG7 1LD, UK; e Diagnostic and Interventional Radiology, The Hospital for Sick Children, Toronto, ON, Canada; f QuSpin Inc., 331 South 104th Street, Suite 130, Louisville, CO 80027, USA; g Centre for Human Brain Health, School of Psychology, University of Birmingham, Edgbaston, Birmingham, UK; h Centre for Rehabilitation and Ageing Research, School of Medicine, University of Nottingham, Nottingham NG7 2UH, UK; i Nottingham Biomedical Research Centre, Nottingham, UK

**Keywords:** Magnetoencephalography (MEG), Optically pumped magnetometer (OPM), Multiple sclerosis (MS), Movement, Beta oscillations, Gamma oscillations

## Abstract

Multiple Sclerosis (MS) is a common neurological disorder in which myelin damage affects neuronal signalling. Magnetoencephalography (MEG – the measurement of magnetic fields generated by neuronal currents) offers metrics of brain function that relate directly to electrophysiological signalling, making it a valuable tool for exploring how abnormal function relates to MS symptoms. However, conventional MEG requires participants to be seated or supine with limited head and body motion. This makes it hard to measure brain function whilst simultaneously asking patients to carry out tasks they find challenging – many of which relate to movement. Here, we used a newly developed OPM-MEG system, with a wearable helmet and a lightweight backpack-mounted control unit, to measure MEG signals in people with MS (pwMS), both at rest and during a visuo-motor task. Uniquely, our system enabled data collection in participants who were seated and standing. We found that established markers of MS – including delayed beta-band responses to finger movement and diminished gamma-band responses to visual stimulation – were measurable using OPM-MEG. Further, we showed that standing (compared to sitting) decreased beta-band connectivity (in patients and controls, but the effect was only significant in controls) and decreased oscillatory power (in patients but not controls). In summary, our paper confirms that OPM-MEG is a useful means to investigate MS; it also demonstrates the importance of investigating how changes in posture relate to oscillations and connectivity, and lays the groundwork for broader studies of movement.

## Highlights

• OPM-MEG is newly developed instrumentation to measure human brain electrophysiology.
• We used OPM-MEG to detect differences between people with MS (pwMS) and controls.
• Measured delayed movement induced beta responses and reduced visual gamma effects.
• OPM-MEG allows recording in participants in multiple postures (sitting/standing).
• We found reduced beta power and functional connectivity when standing (cf. sitting).

## 1 Introduction

Multiple Sclerosis (MS) is a neurological disorder in which damage to myelin in the brain and spinal cord affects neuronal signalling. MS is common (affecting around 1 in every 500 people in the UK (Mackenzie et al., 2014)) and results in wide-ranging and debilitating symptoms including physical disability, cognitive impairment and altered mental health. MS is diagnosed by a combination of symptoms and signs supported by paraclinical examinations, importantly using magnetic resonance imaging (MRI) to reveal lesions which represent demyelination in the white matter. However, white matter lesion load, whilst helping to confirm a diagnosis, correlates poorly with symptoms (Barkhof, 2002). This ‘clinico-radiological paradox’ has led to a search for alternative biomarkers which can improve our understanding of MS pathophysiology, and provide objective metrics to monitor disease progression, inform prognosis and identify appropriate treatments for individual patients. It is now established that structural pathology in MS extends beyond white matter lesions; for example to diffuse white matter damage (Kutzelnigg et al., 2005) and grey matter lesions (Tallantyre et al., 2010) (though both are challenging to measure). It is also clear that myeloarchitecture is closely linked to the brain's electrical function (Helbling et al., 2015, Hunt et al., 2016) and so degradation of myelin and the axonal loss observed in MS should have a marked effect on electrophysiological activity. This means that magnetoencephalography (MEG) (which measures the magnetic fields generated by neuronal current in the brain) should provide important insights into MS pathophysiology. In this paper, we seek to use a new MEG technology to investigate altered neural dynamics in MS patients.

Conventional MEG (Cohen, 1972, Hämäläinen et al., 1993) has been used elucidate differences in electrophysiological function between people with MS (pwMS) and controls (for a systematic review see Khan et al., 2021). Findings broadly fall into four categories: 1) In the resting state (where MEG data are recorded with a patient doing nothing) there is disruption to neural oscillations (the repetitive/rhythmic signals that dominate electrophysiological recordings). E.g., alpha (8–13 Hz) oscillations are shifted in either amplitude or frequency (Kim et al., 2019, Van Der Meer et al., 2013). 2) In somatosensory experiments, phase-locked evoked responses following a sensory stimulus exhibit increased latency (Karhu et al., 1992) compared to controls, in keeping with the role of demyelination in the speed of nerve conduction. 3) MS patients exhibit reduced power or increased latency of induced responses (i.e., changes in neural oscillations in response to a task). Examples include altered beta (13–30 Hz) responses in sensorimotor regions during (or following) movement (Arpin et al., 2017, Barratt et al., 2017, Waldman et al., 2020) and reduced amplitude gamma (>30 Hz) oscillations following visual stimulation (Barratt et al., 2017, Stickland et al., 2019, Waldman et al., 2020). 4) Finally, several studies (e.g., Tewarie et al., 2014a, Tewarie et al., 2014b) have shown altered functional connectivity that correlates better with disability than MRI-based metrics. There remain barriers to the clinical translation of these measures, including the preliminary nature of the results, high inter-subject variance and the high cost and relative scarcity (compared to e.g., MRI) of MEG technology. However, these findings provide support for further studies to develop and validate MEG-based biomarkers for MS which may help solve the clinico-radiological paradox, and provide an objective means of tracking MS progression.

To date, all MEG studies in MS have relied on instruments in which neuromagnetic fields are measured using cryogenically cooled sensors, based on superconducting quantum interference devices (SQUIDS). However, recent years have seen the introduction of optically pumped magnetometers (OPMs) (see e.g., Brookes et al., 2022, Schofield et al., 2022 for reviews). OPMs are small (around the size of a sugar cube), lightweight, and do not require low temperatures. In the context of MS, this offers four potentially important advances. First, OPMs can get closer to the scalp, meaning increased signal (Hill et al., 2024) and spatial precision might offer increased sensitivity to biomarkers. Second, the lack of cryogenics makes systems less complex and lowers cost, which might make the use of MEG in MS more widespread, offering larger datasets to probe clinically relevant effects. Third, OPMs can be positioned anywhere on the body, and can detect signals from other biomagnetic sources, for example simultaneous measurement of the brain and spinal cord (e.g., Mardell et al., 2024). Gathering multiple signals and probing their interaction potentially offers a new window on altered neural signalling in MS. Finally, and perhaps most intriguingly, the lightweight nature of OPMs means that sensors can move with the head. Consequently, scans can be carried out with a participant in multiple postures (e.g., sitting, lying or standing) or carrying out naturalistic tasks involving movement (e.g., Boto et al., 2018, Holmes et al., 2023, O’Neill et al., 2025, Spedden et al., 2025). Whilst MS has wide-ranging symptoms, issues with mobility have a large impact on quality of life (Barin et al., 2018). Problems with standing, balance, stumbling and general clumsiness are often considered early symptoms; walking speed tends to be diminished in patients (Cohen et al., 2014) and gait abnormalities correlate with levels of disability identified by the extended disability status scale (EDSS) (Zanotto et al., 2022). The combination of high performance, lower cost and the opportunity to study neural activity (and connectivity) as patients attempt to carry out tasks that they find difficult, might offer significant advantages for the study of MS. It will likely lead to a new understanding of neural substrates underling symptoms (e.g., poor balance) and could ostensibly offer new clinically relevant biomarkers which, for example, could help track disease progression (something that is currently challenging using MRI).

Although there is high promise, OPM-MEG remains a new technology, and it is important that we validate its use for MS. Here, our aims were three-fold: first, we sought to show that a recently constructed 192-channel OPM-MEG system (Schofield et al., 2024) could be used to record data in pwMS. Second, we aimed to verify that previously observed markers of MS are measurable using OPMs; to this end, we recorded data during a visuo-motor task and analysed both beta- and gamma-band oscillations, hypothesising a delayed beta response in motor cortex following movement (Barratt et al., 2017) and a diminished visual cortex gamma response to visual stimulation (Barratt et al., 2017, Stickland et al., 2019, Waldman et al., 2020) in pwMS. Finally, we recorded data in pwMS and controls in two postures; seated and standing. (Note that this is not a “movement task” per se, but it is something that would be impossible using conventional MEG.) We investigated any changes in beta power and connectivity in the sensorimotor network when standing compared to seated; this was exploratory since previous literature (Ibitoye et al., 2023, Lau et al., 2014, Thibault et al., 2014) suggested no specific hypotheses.

## 2 Methods

### 2.1 Data collection

#### 2.1.1 OPM-MEG system

Our system comprised 64 OPMs (3rd generation; QuSpin Inc. Colorado, USA), each with sensitivity to MEG signals in three orientations (for a complete description, see Boto et al., 2022, Shah et al., 2020). The array therefore allows 192 independent measurements of magnetic field across the scalp surface (i.e., 192 channels). The triaxial sensitivity of each sensor offers advantages for differentiating brain signals from interference and provides more uniform coverage compared to single or dual-axis sensors (Boto et al., 2022, Brookes et al., 2021). The sensors were mounted in 3D-printed helmets (Cerca Magnetics Limited, Nottingham, UK) which allow even distribution across the scalp. Multiple sizes of helmets were used to accommodate different head sizes. The helmets were designed with an open lattice structure to enable efficient convection of heat generated by the sensors.

**Fig. 1:**
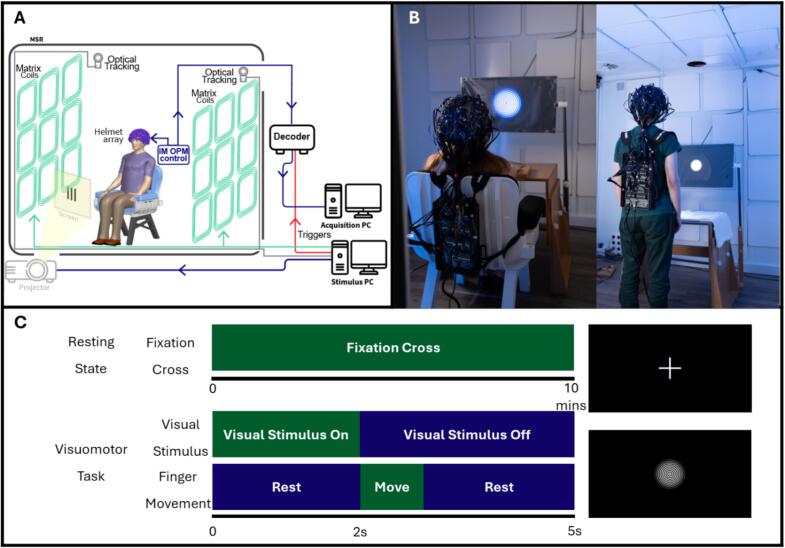
**Experimental set up.** A) Schematic diagram of the OPM-MEG system. B) Photographs of example participants in both the seated and standing positions. (Photographs shown with written consent.) Each OPM sensor is 12.4 x 16.6 x 24.4 mm^3^ and weighs 4 g. 64 OPMs are integrated into a 3D-printed helmet worn by the subject (the total helmet weight is 906 g for the largest size). The electronics unit that controls the sensors is integrated into a backpack, which is 0.36 x 0.2 x 0.06 m^3^ and weighs 1.8 kg. The electronics unit is connected to the outside of the MSR by just two cables, making the system well suited to experiments involving patients who are changing posture or moving naturally. C) A schematic representation of the tasks; resting state in the upper panel and visuo-motor in the lower panel.

The OPM array requires control signals to be sent, and output signals received, independently yet synchronously, to and from each of the 64 sensors. This was enabled via an integrated miniaturised electronics unit (*“NEURO-1”*, QuSpin, Colorado, USA; for a complete description see Schofield et al., 2024). The unit is compact, lightweight, and wearable as a backpack (see Fig. 1B). Each sensor is connected to the backpack via a lightweight ribbon cable. The backpack itself has only two connections to the ‘outside world’ – a power cable and an ethernet connection – the latter sends the digital output from all sensors to a decoder which integrates MEG signals from all channels with other information (for example, relating to stimulus timing) and is connected to a PC, enabling data visualisation and storage. This lightweight and wearable OPM-MEG system is ideally suited to studies in which subjects are asked to change posture or move.

The system was housed inside a 3 m x 3 m x 2.4 m magnetically shielded room (MSR) (MuRoom, Magnetic Shields Limited, Kent, UK) which attenuates both DC and time varying environmental magnetic fields. Importantly, if an OPM is allowed to move in the presence of a static background field (even the small fields that exist inside an MSR) then changing fields will be measured which form artefacts in MEG data (Rea et al., 2021). For this reason, for studies in which people can move, it is important to minimise the static (DC) field in a volume enclosing the head (Boto et al., 2018) (i.e., if sensors move through a background field of zero, then no such artefact will be measured). To achieve this, our MSR was equipped with two systems: First, degaussing coils demagnetise the walls of the MSR; this reduces the remnant static (DC) field to ∼3–5 nT (Altarev et al., 2015). Second, a “Matrix Coil” is used (Holmes et al., 2023); this comprises 96 coil-elements, each of which generates a different field. These fields (which are independently controlled by varying the current through each coil) sum to generate a field at the head location that is equal and opposite to that in the MSR. For this study, the currents were optimised prior to data collection, based on the average DC fields measured throughout the room (Rhodes et al., 2023). Two sets of currents were used, one optimised field surrounding the head for a seated participant, and a second optimised field around the head for a standing participant. In both cases, this resulted in fields < 1 nT in the volume surrounding the head.

In addition to field control systems, the MSR was also equipped with a motion capture system (OptiTrack, Flex13, NaturalPoint Inc., Oregon, USA) which uses an array of six cameras to track the position of infrared *retro*-reflective markers which are attached to the participant.

A single PC controlled the OPM array, data collection and degaussing. A separate PC controlled all stimuli delivered to the participants (see below) as well as the matrix coils and motion capture system. A system schematic is given in Fig. 1A. Photographs of example participants using the system (in the seated and standing postures) are shown in Fig. 1B.

### 2.2 Participants

The study was approved by the UK National Health Service (NHS) Health Research Authority Research Ethics Committee (IRAS Project ID 326694). All participants provided written informed consent prior to taking part. Twenty healthy controls (HCs) were recruited alongside twenty pwMS. This cohort size was informed by previous studies (Barratt et al., 2017, Waldman et al., 2020).

Exclusion criteria were an inability to comply with the study protocol, inability to provide informed consent and pregnancy. Specifically, at screening participants were deemed ineligible if, based on self-report or investigator assessment, they were unlikely to be able to complete the tasks (including standing for a prolonged period) safely. HCs were selected to ensure age and sex distribution were consistent between pwMS and control groups. The study demographic is summarised in Table 1.

**Table 1:** Summary of the demographic characteristics of the study cohort.

	People with MS (N = 20)	Healthy controls (N = 20)
Age (median (IQR))	49.7 (35.7–55.8)	46.3 (33.9–54.3)
Sex (number of females)	14 (70 %)	15 (75 %)
Handedness (number right handed)	18	19
Phenotype (number of relapsing remitting)	18	N/A
Phenotype (number of secondary progressive)	1	N/A
Phenotype (number of primary progressive)	1	N/A
Disease duration (median years (IQR))	6.0 (2.2–14.3)	N/A
EDSS (median (IQR))	2.0 (1.6–3.4)	N/A

#### 2.2.1 Experimental paradigms

All subjects underwent data collection during two experimental paradigms:

• ***Visuomotor experiment****:* In a single trial, visual stimulation was presented in the form of an inwardly moving circular grating ($2.8{}^{\circ}s^{-1}$). The grating was displayed via back projection onto a screen located ∼1 m in front of the subject. The grating was designed to have 100 % contrast and subtend a visual angle of 5.6° (at 1 m distance), with 1.6 cycles per degree. A trial comprised 2 s of simulation followed by a 3-s rest period, during which a grey fixation dot was shown, located centrally on a black background. When the visual stimulus disappeared from the screen, the participant was instructed to make a single abduction and adduction of their right index finger. All participants wore a glove to which five infrared retroreflective markers were attached (one on the index finger, one on the little finger, and three on the back of the hand). This enabled the finger movement to be measured using the motion capture cameras. A schematic representation of the task is shown in Fig. 1C (lower panel). 60 trials were repeated, meaning the experiment lasted 300 s in total.
• Variants of this experiment have been used previously (e.g., Barratt et al., 2017, Hoogenboom et al., 2006, Iivanainen et al., 2020); we expected an increase in gamma band (35–70 Hz) oscillations (relative to baseline) induced by the visual stimulus. We also expected a drop in beta (13–30 Hz) oscillatory amplitude in sensorimotor cortex during movement, followed by an increase (above baseline – termed the beta “rebound”) on movement cessation. In accord with previous literature (Khan et al., 2021), we hypothesised that stimulus-induced gamma oscillations in visual cortex would be diminished, and that the beta rebound in motor cortex would be slower to reach its peak in pwMS compared to controls.
• ***Resting state****:* Participants were asked to relax and “think of nothing” whilst 600 s of OPM-MEG data were acquired (Fig. 1C – upper panel). A fixation cross (a grey dot located centrally on a black screen) was displayed throughout the experiment. Here, we aimed to characterise both resting state oscillatory power and functional connectivity between brain regions.

Both paradigms were repeated twice (i.e., four experiments per participant); once where the participant was seated on a patient support, and once where the participant was standing. For practical reasons, we did not change the height of the visual stimulus between conditions. This meant that the angle at which the visual stimulus was viewed changed across the two repeats of the paradigm. The order of the four experiments was pseudo-randomised across participants. The total time required to carry out all four experiments (including scanner set up) was approximately 45 min.

#### 2.2.2 Co-registration of MEG data to anatomy

Prior to MEG data acquisition, a structured light camera (Einscan H, SHINING 3D, Hangzhou, China) was used to gather a 3D digitisation of the participant’s head. Specifically, we acquired two structured light scans, one without the OPM-MEG helmet and a swimming cap worn by the subject to flatten their hair (the *no-helmet-digitisation*), and a second with the helmet in place (the *face/helmet-digitisation*). We used a “pseudo-MRI” approach (Rier et al., 2024) in which an age-matched T1-weighted template MRI was chosen from a database (Richards et al., 2016) for each participant. The no-helmet-digitisation was then used as a reference and the template MRI warped to fit the digitisation (using the Linear Image Registration Tool (FLIRT) in FSL (Jenkinson et al., 2002, Jenkinson and Smith, 2001)). This technique allows for an approximation to brain anatomy without the need for acquisition of individual MRIs. Following creation of the pseudo-MRI, the head and face surface were extracted (using Fieldtrip (Oostenveld et al., 2011)). We then fitted the helmet/face-digitisation to the face extracted from the MRI (using MeshLab (Cignoni et al., 2008)). Finally, the helmet CAD file was fitted to the face/helmet-digitisation. This locates the sensors relative to the pseudo-MRI anatomy. We chose to use this pseudo-MRI approach, distinct from collection of individual anatomical MRI scans for each subject for two reasons; firstly to reduce the inconvenience to participants (and the overall cost of the study) and secondly because recent work (Rhodes et al., 2025a) suggests that, particularly for group studies, the use of pseudo-MRI works well for OPM-MEG data.

#### 2.2.3 Data collection

For all participants, an experimenter was present in the MSR whilst data were collected; this was to ensure compliance with the tasks and to offer support if, for example, a participant became tired during the standing experiments and wanted to stop the scan or sit down.

After the subject and experimenter entered the MSR, the door was closed, and degaussing was initiated to demagnetise the inner metal walls. The sensor startup procedure (during which the OPM cells are heated and the laser frequency required for optical pumping is locked) was also initiated. Degaussing, and sensor startup took approximately 3 min

Prior to each of the four experiments, matrix coil elements were energised to reduce the DC field in the volume surrounding the subject’s head. In addition, the field within each OPM was “zeroed” and known fields were generated by on-board-sensor electromagnetic coils to calibrate all sensor outputs. In total, these procedures take ∼60 s.

OPM-MEG data were captured throughout each experiment at a sample rate of 375 Hz. The timing of visual stimulation (for the visuo-motor task) was captured via voltages (triggers) which were sent to the OPM-MEG decoder from the parallel port of the stimulus PC; separate triggers marked the onset and offset of visual stimulation.

Motion tracking data (recording finger movement during the visuo-motor task) were acquired simultaneously with OPM-MEG at a sampling frequency of 120 Hz; the motion capture and MEG data were synchronised via a single voltage pulse sent to both systems at the beginning of each experiment.

Throughout the scanning session, the participant and experimenter inside the MSR could contact the scanner operator via an intercom. Scanner operators could also see the participant on a screen, via a camera located inside the MSR.

### 2.3 Data processing

#### 2.3.1 Pre-processing

All data were organised according to the Brain Imaging Data Structure (BIDS) for MEG (Niso et al., 2018). For OPM-MEG data, an infinite impulse response notch filter was applied to remove powerline noise (50 Hz, 100 Hz and 150 Hz). Data were then bandpass filtered into the 1–150 Hz band using a 4th order Butterworth filter. Then a power spectral density plot was created (using Welch’s method (Welch, 1967)) for all channels, and any channels with no signal (e.g., due to a sensor becoming inactive during acquisition) or showing high noise (for example due to a faulty connection to the sensor head) were removed. Finally, homogeneous field correction (HFC) (Tierney et al., 2021) was applied to all data to remove interference that manifests as a spatially uniform field across the OPM array.

#### 2.3.2 Visuo-motor task – measuring the gradient of the beta rebound

We initially aimed to segment the OPM-MEG data into task trials, based on finger movement. To this end, we took the data from the motion capture system and up-sampled (using linear interpolation) to 375 Hz. We then isolated the three signals representing the x, y, and z coordinates (in the coordinate system of the cameras) of the tip of the participants right index finger. Each of these signals was differentiated to give finger velocity, and the three signals combined in quadrature to provide a single measure of finger speed as a function of time. To remove noise, a 7-Hz low pass filter (4th order; Butterworth) was applied. For every finger abduction (i.e., every trial) there were two clear peaks in finger speed – the first where the index finger moves away from the other fingers (abduction), and the second where it moves back (adduction). These two peaks were identified. We then found the first local minimum in the signal immediately following the second peak and used this to denote the point at which the finger stops moving (designated as t = 0, for each trial).

The OPM-MEG data were segmented into 5-s trials (−1 s < t < 4 s relative to movement cessation). Any bad trials (defined as an individual trial in which the signal variance (at any one channel) exceeds three times the standard deviation of variance measured across all trials (for that channel)) were removed. All trials were also inspected visually and trials with high noise levels were removed.

We used a linearly constrained minimum variance (LCMV) beamformer (Robinson & Vrba, 1998) to construct pseudo-T-statistical images showing the spatial signature of task induced change in oscillatory power in the beta band. Specifically, the data were filtered to the 13–30 Hz band. A data covariance matrix was constructed using data from the whole experiment (excluding bad trials) and Tikhonov regularisation applied (using a regularisation parameter equal to 5 % of the maximum eigenvalue of the unregularised matrix (this is the case for all covariance matrices used in this paper)). Beamformer weights were constructed using the regularised data covariance and a forward solution based on a single shell volume conductor model ((Nolte, 2003), implemented using FieldTrip). To make the pseudo-T-statistical image, we contrasted projected oscillatory power in the −1 s to 0 s (active) window to the 0.5 s to 1.5 s (control) window (timings all relative to movement cessation). Pseudo-T-statistics were derived for voxels on a regular 4-mm grid covering the whole brain. At each location, source orientation was determined as the direction of maximum beamformer projected signal amplitude (Sekihara et al., 2004). These images were normalised (to ensure that no single participant dominated the group result) and averaged across participants within each group (pwMS/controls) and for each posture (seated/standing) for visualisation.

For the location showing the largest movement induced beta modulation (derived individually for each subject using the pseudo-T-statistical images as used by Barratt et al., 2017), we generated a time–frequency spectrogram (TFS). (Note, these peaks were always in sensorimotor cortex.) Beamformer weights were derived using covariance calculated in the 1–150 Hz band. These weights were then used to derive a broadband estimate of electrophysiological activity at the location of interest (termed a virtual electrode (VE)). VE data were frequency-filtered into overlapping bands between 1 and 120 Hz (specifically: 1–4 Hz; 2–6 Hz; 4–8 Hz; 6–10 Hz; 8–13 Hz; 10–20 Hz and then 10 Hz wide bands, overlapping by 5 Hz, (i.e., 15–25 Hz; 20–30 Hz etc) up to 120 Hz) and, for each band, a Hilbert transform was used to derive the analytic signal. The absolute value of the analytic signal was then calculated to generate the instantaneous amplitude of the band-limited signals – termed the Hilbert envelope. This was averaged over trials to give a single, trial averaged, time course of oscillatory amplitude per frequency band, A(t,f). For all frequency bands, we calculated a baseline oscillatory amplitude, B(f) in the 3 s ≤ t ≤ 4 s time window relative to the offset of movement. This baseline, at the end of the trial, was chosen during a period of no finger movement and was similar to the baseline chosen by Barratt et al., 2017. We then computed the final TFS as $R\left(t,f\right)=\left(A\left(t,f\right)-B\left(f\right)\right)/B\left(f\right)$, which represents the relative change in oscillatory amplitude for all frequencies. In addition to the TFS, we derived the beta amplitude envelope in sensorimotor cortex; here the VE was computed using only beta-band filtered data and the Hilbert envelope was calculated and averaged over trials. Again, data were averaged across participants in each group, and for each posture.

Our hypothesis, based on previous work by Barratt et al., 2017 was that the post-movement beta rebound would be slower to rise in pwMS compared to controls. To test this statistically we used a permutation test (Maris & Oostenveld, 2007). For each group, we measured a best fit of the gradient of the average envelope in a time interval corresponding to the time points at which beta power showed a relative amplitude of 0 % and 75 %, relative to the maximum amplitude of the rebound (i.e., this is the time window when the beta rebound is increasing). The difference in gradients between groups was then computed. To test significance, we calculated the same gradient difference in groups comprising randomly selected participants (i.e., “sham” groups, with the same number of participants, but containing random mixtures of patients and controls). This was repeated 20,000 times and a null distribution was generated. We compared the real value to the null distribution and an empirical p-value was derived. This test was repeated for data recorded in the two different postures.

#### 2.3.3 Visuo-motor task – gamma oscillations

We used a beamformer to characterise the spatial, temporal and spectral properties of gamma oscillations induced by the visual stimulus. Here, data were again segmented into 5-s trials, but relative to the onset of visual stimulation (rather than the offset of movement; i.e., t = 0 represents the start of the visual stimulus, not the offset of movement as was the case for the beta band analysis). Data were filtered into the 35–70 Hz band and pseudo-T-statistical images were derived as described above, but contrasting gamma power in the 0 s to 2 s (active) window during stimulation, to the 3 s to 5 s (control) window during no visual stimulation (timings all relative to stimulus onset). These windows were chosen as an increase in gamma amplitude is expected for the full duration of the stimulation and gamma amplitude falls to baseline at the offset of stimulation (Hoogenboom et al., 2006). TFS and the time course of the envelope of gamma oscillations were derived at locations corresponding to the group mean maximum of gamma increase. A baseline window of 4–5 s (where no visual stimulation was presented) was used. Here we used the group mean location, rather than maxima for each participant, as not every participant had a gamma response. (This is a similar approach to that used by Waldman et al., 2020.)

Here, our hypothesis was that stimulus induced gamma amplitude change would be diminished in patients relative to controls. To test this, we again used a permutation test. We took group averaged data and measured the difference in gamma amplitude from baseline (in the 0 s to 2 s window) between patients and controls; we then compared this value to a null distribution; we calculated the same amplitude difference between groups comprising randomly selected mixtures of patients and controls. This was repeated 20,000 times, a null distribution created and an empirical p-value derived. This test was again repeated for data recorded in the two different postures.

#### 2.3.4 Resting state oscillatory power in the beta band

To remove artefacts from the resting state recording, the data were segmented into 60, 10-s segments. Any bad segments (with high noise) were identified by visual inspection and removed. Following this we applied independent component analysis (ICA) (using Fieldtrip (Oostenveld et al., 2011)) to remove components that related to eyeblink and cardiac artefacts.

We aimed to characterise oscillatory power across the cortex. To this end, the brain was first parcellated into 82 cortical regions, defined by the MarsAtlas (Auzias et al., 2016). This was achieved by first defining the MarsAtlas in the space of the MNI template (ICBM152) brain. This template was then warped to the brain of every individual participant using FLIRT in FSL (Jenkinson et al., 2002, Jenkinson and Smith, 2001). The same transform was then applied to the MarsAtlas to define the 82 regions in each individual’s anatomical space. The coordinates of the centre of mass (centroid) of each region were then determined.

A VE was constructed for each centroid using a beamformer: The 1–150 Hz filtered data were used to generate covariance matrices, constructed using data recorded throughout the whole experiment (excluding bad segments). The forward model (again based on a single shell volume conductor model) and data covariance were then used to calculate VE timecourses for each region of the MarsAtlas (source orientation was determined as the direction of maximum projected signal amplitude).

For each region, we took the broadband beamformer projected data and used Welch’s method to estimate the broad-band power spectral density (PSD). In addition, we frequency filtered the VE data to the beta band (4th-order Butterworth filter) and once again derived a PSD. We then computed the area under the beta-band PSD, normalised by the area under the broadband PSD, to give a measure of “*relative beta power*” (i.e., the fraction of the total spectrum that exists in the beta band) (Lew et al., 2021, Rier et al., 2023). This was repeated for all brain regions in the MarsAtlas and plotted as a functional map. (Note that we restricted our analyses to the beta band due to the strong link between beta oscillations and movement (Pfurtscheller & Lopes da Silva, 1999); however an equivalent analysis for alpha activity is presented in the appendix.)

We aimed to test two exploratory hypotheses related to beta power: 1) *That standing, compared to sitting, changes beta amplitude in the sensorimotor network*. To test this, we averaged the relative beta power across all regions of the sensorimotor network, for all participants, for both postures. The sensorimotor network was chosen as we reasoned it might show the largest difference in power between sitting and standing. The regions included in the network were the Ventral Somatosensory Cortex, Dorsolateral Somatosensory Cortex, Dorsomedial Somatosensory Cortex, Ventral Motor Cortex, Dorsolateral Motor Cortex and Dorsomedial Motor Cortex, for both hemispheres, as defined by the MarsAtlas (Auzias et al., 2016). We then used a Wilcoxon sign rank test to assess whether the difference in relative beta power between conditions (sitting and standing) was derived from a distribution whose median was non-zero; this test was carried out for pwMS and controls independently (i.e., two separate statistical tests). 2) *We hypothesised that relative beta power would differ between pwMS and controls*. Here, we again averaged relative beta power over sensorimotor regions and used a Wilcoxon sum-rank test to determine whether any difference between patients and controls was significant. This test was carried out for seated and standing conditions independently (i.e., two further statistical tests). We then Bonferroni corrected the threshold for significance to account for the four statistical tests carried out.

#### 2.3.5 Resting state functional connectivity in the beta band

We also aimed to characterise functional connectivity between all pairs of regions in the MarsAtlas. This was achieved using amplitude envelope correlation (AEC) (Brookes et al., 2011, O’Neill et al., 2015). Beamformer projected beta-band filtered resting state data were generated for all 82 centroids. For each pair of brain regions, pairwise orthogonalisation was applied to reduce the effect of source leakage (Brookes et al., 2012, Hipp et al., 2012). Following this, the Hilbert envelopes for the two regions were calculated. These envelopes were down-sampled temporally from 375 Hz to 75 Hz and the Pearson correlation coefficient between the envelopes was calculated to quantify functional connectivity. This was applied to all ((82^2^ – 82)/2 = ) 3321 region pairs, resulting in a whole-brain connectome matrix showing how each region is connected to every other region.

The AEC was computed independently for each posture and each participant separately, and the resulting matrices averaged over participants in each group and for each posture, for visualisation. In addition, matrices were thresholded to keep only the 266 strongest connections, and these were plotted as lines within a glass brain, for visualisation. We also calculated connectivity strength for each region (i.e., how connected a region is to every other region – calculated as the sum of all elements along a row (or column) of the matrices). The summed connectivity strength across all regions in the sensorimotor network (termed *sensorimotor connectivity strength*) was then computed.

We aimed to test two exploratory hypotheses relating to functional connectivity: 1) *That standing, compared to sitting, changes beta connectivity in the sensorimotor network*. To achieve this, we took the sensorimotor connectivity strength metrics for the two conditions and used a Wilcoxon sign rank test to assess whether any difference in beta connectivity was significant; this was carried out for patients and controls separately. 2) *That sensorimotor connectivity strength differed in patients compared to controls*. Here, we took sensorimotor network connectivity and used a Wilcoxon sum-rank test to determine whether any difference between patients and controls was significant. This was carried out for seated and standing conditions independently. Again, we used Bonferroni correction to account for multiple comparisons.

## 3 Results

The OPM-MEG system was acceptable to the cohort, with 40 participants completing the scanning session. For the beta-band analysis (visuomotor task), 3 datasets from each group were removed due to a failure of the software that controlled the motion tracking system (meaning no motion tracking data were collected, and consequently we were unable to accurately determine the timing of the finger movement). This resulted in 34 datasets in the final beta-band analysis of the visuomotor task. For the gamma band analysis of the visuomotor task and the resting state task (where movement data were not required), all 40 datasets were included.

On average we removed 16 ± 7 channels for the visuomotor task and 12 ± 8 channels for the resting state task (mean and standard deviation across all pwMS and all controls for the beta- and gamma-band analyses, for seated and standing conditions). This was due to either high noise or no signal. We removed 10 ± 8 trials from the visuomotor task and 10 ± 11 segments from the resting state analyses due to high noise. (For a complete breakdown of how these values varied across groups, tasks and conditions, see Supplementary Material, Table S1.)

### 3.1 Visuo-motor task

**Fig. 2:**
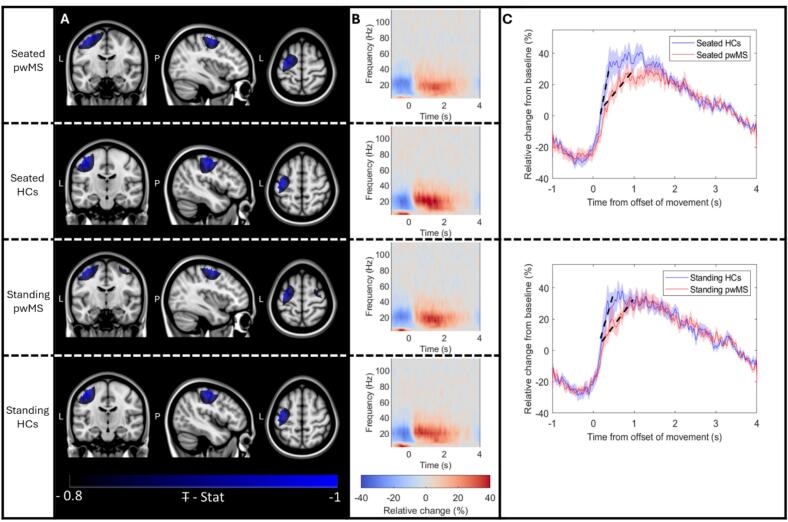
**Beta-band modulation with finger movement.** A) Group averaged pseudo- Ŧ-statistical images (normalised by the largest deflection from zero) showing the location of the beta modulation with finger movement (blue) overlaid on a standard brain. B) Time frequency spectra showing the modulation of oscillatory amplitude in the 1–120 Hz band, as a function of time, averaged across trials. Red represents a relative (%) increase in oscillatory amplitude and blue represents a decrease, compared to baseline. In A and B, the upper panel shows seated pwMS, the upper-centre panel shows seated controls, the lower-centre panel shows standing pwMS, and the lower panel shows standing controls. C) Relative change in beta band (13–30 Hz) amplitude; pwMS are shown in red, controls in blue; dashed line shows the gradient; shaded areas represent standard error over subjects. The upper panel represents seated data, and the lower panel shows standing data. In panels B and C, the baseline window was 3 s < t < 4 s. Note that the rate of increase of the rebound is significantly different in pwMS relative to controls for the seated but not the standing experiments.

Fig. 2 shows results from the beta-band analysis of the visuomotor task. Fig. 2A shows pseudo-T-statistical images, representing the spatial signature of beta modulation (in blue), overlaid on the standard brain. Images have been normalised and averaged across participants. Fig. 2B shows group averaged time frequency spectra taken from the sensorimotor cortex. Red represents an increase in oscillatory amplitude, and blue represents a decrease; both are shown as a fractional change from baseline, where the baseline is calculated in the 3 – 4 s window. Time zero corresponds to movement offset (derived from motion tracking). In both A and B, the upper plots show seated pwMS, the upper-centre plots show seated controls, the lower-centre plots show standing pwMS, and the lower plots show standing controls. In both conditions, and for both groups, the locations of maximum beta modulation localised to the left primary sensorimotor region and the time frequency spectra show clear decreases in beta amplitude during movement with a rebound following movement offset.

Fig. 2C shows the trial averaged Hilbert envelope of beta oscillations for the seated (upper panel) and standing (lower panel) postures (from the same locations of maximum beta modulation as in Fig. 2B). In both panels, the amplitude reduction during movement and the rebound can be seen clearly for both groups. However, in the seated condition there was a significant (p = 0.018; permutation test) difference in the gradient of the beta rebound (marked as a dashed line), which was smaller (i.e., the rebound was slower to rise) in pwMS compared to controls. Whilst the same trend is apparent in the standing data, the effect was not significant. This finding is in good agreement with previous work on MS patients using conventional MEG (Barratt et al., 2017, Khan et al., 2021), supporting the validity of OPM-MEG for detecting MS-related changes in beta-oscillations.

**Fig. 3:**
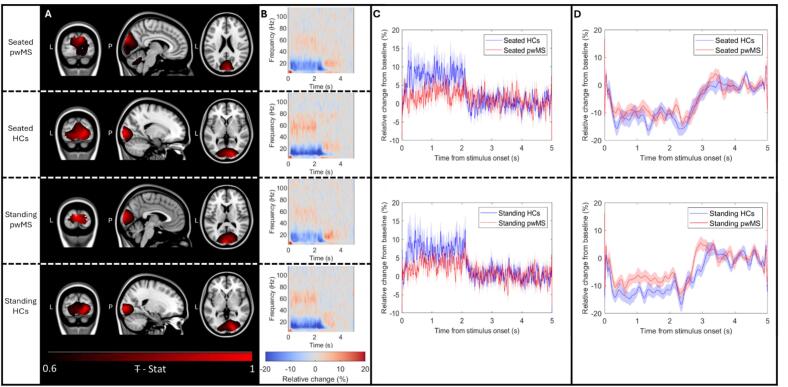
**Neural oscillatory modulation with visual stimulation.** A) Group averaged pseudo-T-statistical images (normalised by the largest deflection from zero) showing the location of largest gamma amplitude change with visual stimulation (red) overlaid on a standard brain. B) Time frequency spectra showing the modulation of oscillatory amplitude in the 1–120 Hz band, as a function of time, averaged across trials and participants. In both A and B, the upper panel shows seated pwMS, the upper-centre panel shows seated controls, the lower-centre panel shows standing pwMS, and the lower panel shows standing controls. C) Relative change in gamma band (35–70 Hz) amplitude; pwMS are shown in red, controls in blue. The upper panel represents seated data, and the lower panel shows standing data; shaded areas represent standard error over subjects. In pwMS the gamma modulation is less pronounced; this was not significant in the two groups independently, but did reach significance using a combined analysis (p = 0.019; permutation test). D) Relative change in alpha band (8–13 Hz) amplitude. Again the upper panel represents seated data; the lower panel shows standing data; pwMS in red; controls in blue; shaded areas represent standard error over subjects. In panels B, C, and D the baseline window was 4 s < t < 5 s.

Fig. 3 shows gamma (35–70 Hz) band oscillations induced by the visual stimulus. Fig. 3A shows spatial signature of the induced gamma increase (in red), overlaid on the standard brain. Fig. 3B shows the associated time frequency spectra (data are again shown as a fractional change from baseline, where the baseline window is 4–5 s. Recall that time t = 0 now represents the onset of the visual stimulus). In panels A and B, the upper plots show seated pwMS, the upper-centre plots show seated controls, the lower-centre plots show standing pwMS, and the lower plots show standing controls. In all cases, data have been averaged across 20 participants (the pseudo-T-statistical images were normalised prior to averaging). In all four cases, gamma modulation localised to the visual areas and the time frequency spectra show a clear increase in gamma amplitude during stimulation (0 s < t < 2 s).

Fig. 3C shows the Hilbert envelope of gamma oscillations for the seated (upper panel) and standing (lower panel) postures. Red shows pwMS and blue shows controls. The induced gamma amplitude was lower in pwMS than controls. This effect did not reach statistical significance when the standing and seated data were treated independently. However, when data from the two postures were averaged and a single statistical analysis performed, the change was significant (p = 0.019; permutation test).

A change in the alpha band response to the visuomotor task, between pwMS and controls was not in the initial hypotheses for our study. Nevertheless, when examining the data we noticed that the stimulus induced reduction in alpha oscillations appeared more pronounced in controls than pwMS. We therefore conducted a post-hoc statistical test. Fig. 3D shows the Hilbert envelope of alpha (8–13 Hz) oscillations for the seated (upper panel) and standing (lower panel) postures. Red shows pwMS and blue shows controls. The mean difference in alpha amplitude between pwMS and controls was measured in the 0 s to 2 s window during visual stimulation (as was used for gamma band analysis) and statistical significance determined using a permutation test. Results showed that the difference between pwMS and controls was not significant.

### 3.2 Resting state results

**Fig. 4:**
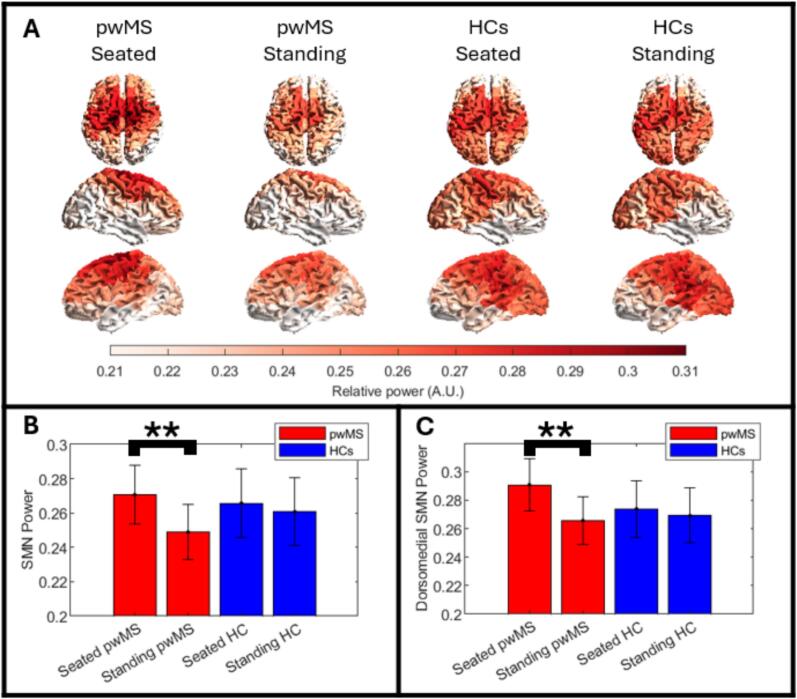
**Resting state beta power, seated and standing.** A) Group averaged maps showing the spatial distribution of relative beta power (calculated as the area under the spectral curve in the beta-band (13–30 Hz), divided by the total area under the spectral curve for all frequencies (1–150 Hz)). The brain has been parcellated according to the MarsAtlas and, as expected, the largest relative beta power is found in the sensorimotor regions for both pwMS and controls. B) Bar chart showing beta power averaged across all regions in the sensorimotor network, for pwMS and controls, seated and standing. In all cases the height of the bar represents a mean and the error bar represents standard error across subjects. C) Equivalent to B, but results are only shown for the dorsomedial sensorimotor cortex. ** denotes a significant (p < 0.05) change after multiple comparison correction.

Fig. 4A shows the spatial distribution of resting state beta power, measured as the fraction of the total oscillatory power in the beta-band; higher fractional power is shown in red, lower fractional power in white. The far-left plots show seated pwMS; centre left shows standing pwMS; centre right shows seated controls, and far-right shows standing controls. In all four cases, results are averaged across 20 participants. The largest fractional beta power is observed over the sensorimotor regions with the overall distribution consistent with previous findings (e.g., Lew et al., 2021, Rier et al., 2023). This was the case regardless of posture or group. The smallest fractional beta power appeared in the temporal lobes – however, this could be due to a lack of coverage in this region.

Fig. 4B shows the summed beta power across all regions of the MarsAtlas that fall within the sensorimotor network. Values for both postures and both groups are shown; the height of the bar indicates the mean and the error bar represents standard error, across participants. Interestingly, there was a significant (p = 0.003; Wilcoxon sign-rank test) decrease in fractional beta power in pwMS, when moving from sitting to standing, but this difference was not observed in control participants. There was no observable difference between pwMS and controls in either the seated or standing resting state measurements. Fig. 4C shows the same thing as Fig. 4B, but here, only relative beta power for the dorsomedial sensorimotor network is shown (i.e., the regions covering the leg and foot areas of the sensorimotor representations, which we assumed were the most likely areas for power change in a standing task). Results are similar in showing a significant (p = 0.005; Wilcoxon sign-rank test) drop in beta power in pwMS when standing compared to sitting, but no equivalent drop in controls and no difference between pwMS and controls.

**Fig. 5:**
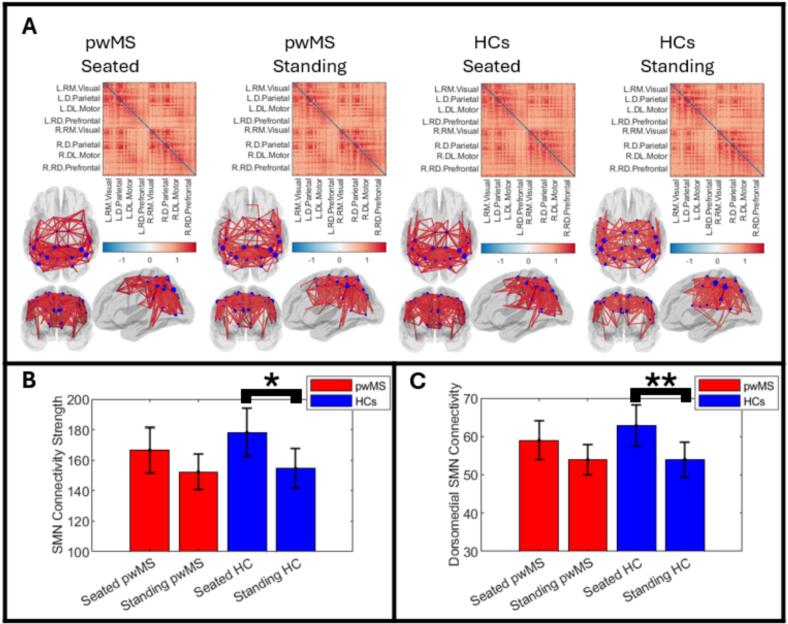
**Resting state beta connectivity, seated and standing.** A) Connectome matrices, and corresponding glass brain plots, showing patterns of beta band functional connectivity for pwMS and controls, seated and standing. The glass brain plots show the 266 connections between regions in the MarsAtlas with the largest functional connectivity. The blue dots represent connectivity strength for each region (i.e., how connected that region is to every other region in the brain). Notice that the largest connections appear in the sensorimotor and posterior parietal regions as expected. B) Bar chart showing beta connectivity strength summed across the sensorimotor network, for pwMS and controls, seated and standing. The height of the bar represents the mean and the error bar shows standard error across subjects. C) Equivalent to B, but results are only for the dorsomedial sensorimotor cortex. ** denotes a significant (p < 0.05) change; * indicates a trend that did not survive multiple comparison correction.

Fig. 5A shows resting state connectome matrices; here each matrix element represents a single value of pairwise functional connectivity between two brain regions, with darker red indicating a stronger connection. The far-left plots show seated pwMS; centre left shows standing pwMS; centre right shows seated controls, and far-right shows standing controls. Results are averaged across 20 participants. The glass brain plots show the 266 strongest connections (plotted as red lines between regions). The diameter of the blue circles represents connectivity strength (i.e., how connected that region is to all other regions in the brain). These resting state beta networks show the strongest connections between bilateral posterior parietal regions as well as throughout the sensorimotor network. Fronto-parietal connections within each hemisphere are also visible. This pattern is in agreement with those observed previously (Hunt et al., 2016).

Fig. 5B shows the summed connectivity strength across all regions of the sensorimotor network. Fig. 5C shows the same thing but for the dorsomedial sensorimotor regions. In both panels, the height of the bar indicates the mean connectivity strength, and the error bar represents standard error across participants. In pwMS and controls, there was a drop in connectivity when standing compared to sitting, but this only reached significance in controls, and only when measured in the dorsomedial regions (Fig. 5C; p = 0.012; Wilcoxon sign-rank test). A similar trend was observed across the whole sensorimotor network (Fig. 5B) but this failed to reach statistical significance following multiple comparison correction (p = 0.016, but this does not reach the threshold for significance (0.05/4 = 0.0125); Wilcoxon sign-rank test). (Note: if one combines pwMS and controls into a single group (i.e., 40 participants) and measure the difference between sitting and standing, this is significant (p = 0.015; Wilcoxon sign-rank test over all sensorimotor regions).) We found no significant difference between pwMS and controls.

## 4 Discussion

There are several signatures of MS that are measurable using MEG, including changes in resting state neural oscillations, delayed evoked responses, reduced power or delayed latency of task induced oscillatory responses and disrupted functional connectivity. These measures enhance our understanding of the pathophysiology of MS and could (in the future) offer a solution to the clinic-radiological paradox, provide routes to monitoring disease progression and help evaluate the effects of treatment (e.g., via longitudinal scanning of patients) (Khan et al., 2021). However, MEG research in MS is limited by the high cost and scarcity of scanners (compared to e.g., MRI). If we could develop a new generation of MEG instrument that is lower cost, this would enable wider deployment for longitudinal monitoring. Moreover, although the symptoms of MS are wide ranging, many patients experience difficulties in standing, moving and walking. Conventional MEG requires that patients remain seated or supine, with limited head motion while data on brain function are acquired; this prevents functional assessment whilst patients attempt to carry out tasks they find difficult. Allowing MEG acquisition while patients carry out such tasks could facilitate the discovery of new biomarkers that relate more directly to symptoms. It might also provide objective metrics to monitor disease progression (before the development of brain atrophy). In this paper we have taken a first step along this path, by demonstrating that OPM-MEG can 1) be used in a clinical population of pwMS; 2) detect known electrophysiological abnormalities, and 3) assess brain function with participants in multiple postures.

Our first aim was to demonstrate that a newly developed OPM-MEG system was able to gather data in pwMS in multiple postures. Our system incorporated a lightweight (906 g) helmet that allowed motion of the head whilst MEG data were acquired. The system also incorporated a lightweight (1.8 kg) miniaturised (0.36 x 0.2 x 0.06 m^3^) electronics control unit worn as a backpack (Schofield et al., 2024) with just two cables directed outside the MSR. This was easily supported by all pwMS, even when standing for a long period. It is noteworthy that previous iterations of OPM-MEG (e.g., Rhodes et al., 2023, Rier et al., 2024) have also used a lightweight wearable helmet which allows head motion. However, each sensor was wired to a control unit (outside the MSR) individually, resulting in 64 cables (one for each OPM) trailing from the helmet. The weight of this cabling would be a challenge for some groups (including pwMS) to support when standing, and so studies were limited to seated participants (albeit with free head motion). The new electronics unit avoids such cumbersome cabling and affords significant advantages when scanning individuals who are standing. A second key enabler to imaging patients in multiple postures was our matrix coil. Any movement through a non-zero background magnetic field creates artefacts in OPM-MEG data. Indeed, even in cases where subjects are notionally still, small movements (e.g., the motion of the head due to the heart beating) can generate artefacts larger than brain signals. Thus, creating a region of low field (<1 nT) in a volume surrounding the head is a useful means to minimise such artefacts (Rea et al., 2021). Previous iterations of OPM-MEG have employed bi-planar coils for this purpose (e.g., Holmes et al., 2018, Jas et al., 2025). However, this only allowed field generation with the head in a single position (seated). The operation of a matrix coil allows field nulls to be generated with the head in multiple positions within the MSR (Holmes et al., 2023), helping to minimise movement artefact regardless of posture. In sum, the wearable helmet, backpack-mounted electronics, minimal cabling and matrix coil combined to make the measurements in the present paper practical. Although the experiments here are simple (i.e., just standing or sitting), this paves the way for more expansive naturalistic experiments, for example stepping, turning, walking or balancing.

The second aim of our paper was to demonstrate that data from our system are of sufficient quality to replicate previously reported (conventional MEG) findings in pwMS. The movement-induced beta-band amplitude reduction, followed by the post-movement beta rebound (Pfurtscheller & Lopes da Silva, 1999) is one of the most robustly observed neurophysiological signals. Recent studies (see e.g., Barone & Rossiter, 2021 for a review) suggest that the rebound provides a top-down inhibitory influence on the primary motor cortex and reinitiates a “status quo” in those regions following movement. Further studies suggest that this top-down signal arises from other areas of the broader sensorimotor network (e.g., Tewarie et al., 2019), suggesting that the signal relies on efficient connectivity between brain regions. Given this, it is perhaps not surprising that in MS – where white matter integrity is diminished – we observe a beta rebound that is slower to reach its peak in pwMS compared to controls. Abnormalities in the beta response to movement have been observed multiple times in pwMS (Arpin et al., 2017, Barratt et al., 2017, Waldman et al., 2020) and the finding we present (Fig. 2) is not new. However, to our knowledge this is the first time that such a finding has been elucidated using OPM-MEG.

We also measured a diminished gamma-band response to visual stimulation in pwMS compared to controls (Fig. 3). This is in agreement with similar measurements made by Barratt et al., 2017 and Waldman et al., 2020. The reduced gamma effect in MS can be explained through the disruption of the minicolumn microcircuit organisation by demyelination, axonal damage and cortical atrophy. These lead to microstructural changes which have been detected by diffusion tensor imaging of the cortex (McKavanagh et al., 2019). Cortical demyelination is also known to cause synaptic loss (Möck et al., 2021) as well as loss of parvalbumin interneurons (Magliozzi et al., 2021, Zoupi et al., 2021) (which are closely associated with the generation of gamma oscillations (Shaw et al., 2017)). These factors collectively impair the synchronization and connectivity required for generating gamma oscillations. It is well-known that the gamma-band response to visual stimulation exhibits marked variation between individuals (Muthukumaraswamy et al., 2010). Importantly, this between subject variance is not “noise”; rather, the visual gamma response measured multiple times within a single individual is highly stable (Muthukumaraswamy et al., 2010). An important clinical aim in MS is to develop methods that track disease progression and, whilst the gamma response may be variable across individuals, its stability within a single person suggests it could be useful in longitudinal studies of minicolumn function, possibly detecting early loss of synapses and interneurons. Such a marker could be made more specific via biophysical modelling approaches which are able to derive direct markers of neural signalling using microcircuit models (e.g., Rhodes et al., 2025b; Shaw et al., 2017).

The final aim of our paper was to show that OPM-MEG offers something new in its ability to probe the brain activity of pwMS in multiple postures. Using our visuomotor task we showed that the task induced changes in beta and gamma band oscillations followed similar trends regardless of posture. This suggests that data quality were maintained across both the sitting and standing sessions. In addition, we measured resting state beta-band oscillatory power and functional connectivity in pwMS and controls, whilst both seated and standing. In the control group, across both postures, beta power was maximal in brain regions associated with movement and extended across parietal and occipital regions. These findings agree with previously reported results (e.g., Mellem et al., 2017; Niso et al., 2019; Hunt et al., (2016)). The distribution of beta power in pwMS was less spatially widespread; whilst this was not necessarily expected, a similar pattern was reported in previous work (Afnan et al., 2023) which compared controls with people with epilepsy. Beta connectivity measures highlighted a network including sensorimotor, bilateral posterior parietal, temporoparietal and frontoparietal connections. These networks were similar in pwMS and controls, and have been highlighted previously using both OPM-MEG (Boto et al., 2021, Rier et al., 2023) and conventional MEG (e.g., Hunt et al., 2016). This again provides confidence that we can gather high quality data regardless of posture.

Interestingly, across the entire cohort (pwMS and controls combined) we found significantly reduced functional connectivity within the sensorimotor network in participants standing compared to sitting. This is in keeping with an EEG finding (Lau et al., 2014) showing that connections involving sensorimotor regions were weaker for walking than standing. Similarly, during simple finger movements, connectivity in the primary motor areas tends to reduce (albeit using phase-based connectivity measurements) (Tewarie et al., 2019). It is therefore tempting to speculate that some regions within the sensorimotor system drop out of the wider network to support standing/movement and this leads to the observable drop in connectivity. When considering the patient and control groups separately, the change in connectivity between seated and standing only reached significance in the control group, whilst the change in beta power between seated and standing was only significant in pwMS. This suggests that beta power is not driving the changes in connectivity observed due to altered signal-to-noise ratio. Rather this appears to be revealing differences, between pwMS and controls, in the way in which neural assemblies engage when standing. It remains to be seen if, in conditions like MS where connectivity is compromised, these reductions significantly impact standing ability. This possibility could be valuable for developing clinical assessments and rehabilitation strategies. What is certain is that our results demonstrate how changing posture affects network connectivity, and future studies – powered by OPM-MEG instrumentation – should investigate this further.

There are some limitations of our study that should be understood. Firstly, our participant cohort was small; whilst sufficient to test the novel equipment, the statistical power to mine our data for effects in the seated and standing postures was limited. Future studies should therefore employ our data (which are publicly available – see below) as a ‘hypothesis generator’ to inspire new studies of how changing posture affects measurable brain activity. Our study was also limited as we only scanned participants seated and standing – thus the study relates to changes in posture and not movement. Nevertheless, we demonstrated that our system could work in multiple locations inside the MSR. This paves the way for future studies of more expansive movements. For example, it is easy to conceive how the experiment could be expanded to a study where subjects change posture (e.g., a “get up and go task”) which has already been demonstrated (albeit in a single healthy participant) using the same OPM-MEG system (Schofield et al., 2024). Such tasks have clinical utility. Similarly, a study where participants turn (e.g., through 90 degrees), march on the spot, or step forwards and backwards, could be a natural expansion. Walking is more difficult due to the confines of the MSR. However, it is not inconceivable that a treadmill could be installed which would allow simultaneous measurement of brain activity and gait. Here, we also made limited use of motion tracking, using it primarily to track finger movement in the visuomotor task. However, motion tracking cameras are capable of vastly more and future studies of posture and movement should exploit this, for example via measurement of postural sway. This is a known marker of cortical dysfunction in a range of conditions, and correlating sway with brain function could represent an interesting avenue of research. Finally, only one of the pwMS selected for this study had a higher EDSS score (6.0), meaning the levels of disability across our cohort were small compared to that generally found in a population of pwMS. This means that we have successfully measured differences between pwMS relative to controls in a population where the effect of the disease is small. Future assessment of a cohort with a higher mean EDSS score may lead to exaggerated effect sizes.

OPM-MEG is not the only means to capture brain function from participants in multiple postures or moving naturally, and this warrants further discussion. EEG has significant clinical utility; it offers a direct, non-invasive and wearable measure of electrophysiology, and (unlike MEG/OPM-MEG) it does not require magnetic shielding. Indeed, fully ambulatory EEG systems are widely available and allow capture of electrophysiological data during almost any imaginable experiment. However, EEG is more susceptible than MEG/OPM-MEG to artefacts from non-brain sources – particularly muscles during movement (Boto et al., 2019, Claus et al., 2012, Muthukumaraswamy, 2013) and this limits sensitivity – particularly to high frequency signals, and when subjects move freely. Such movement artefacts are difficult to disentangle from brain data. EEG also has lower spatial resolution than OPM-MEG and there are two reasons for this. Firstly, EEG data are distorted spatially by the inhomogeneous conductivity of the brain/skull/scalp; this makes EEG signals harder to model than MEG signals which pass through the skull relatively undistorted, and leads to EEG having lower spatial resolution compared to conventional MEG (Baillet, 2017). Secondly, at a fundamental level the ability to resolve two sources in the brain depends on the correlation between the sensor space field patterns generated by those sources. This correlation, in turn, depends on how spatially diffuse the patterns of electrical potential or magnetic field are. For EEG, patterns of electrical potential are made diffuse by the spatial spread of the electrical signal due to the high resistivity of the skull. In conventional MEG, magnetic field patterns are made diffuse because the distance from the scalp to the sensors is large (to accommodate a thermally insulating gap, protecting the head from the cryocooled sensors). (For these reasons, if the modelling difficulties could be overcome, EEG can (in theory) achieve a similar spatial resolution to conventional MEG.) Conversely, OPM-MEG sensors get closer to the scalp and signals are less distorted by the skull; meaning magnetic field patterns are more focal and correlation between field patterns from multiple sources is lower than in either EEG or conventional MEG. This gives OPM-MEG fundamental advantages (compared to conventional MEG and EEG) in terms of spatial resolution (Boto et al., 2019). This said, recent work (e.g., Boto et al., 2019; Seedat et al., 2024) has shown that simultaneous capture of OPM-MEG and EEG is possible. This potentially offers advantages for spatial resolution and sensitivity compared to either modality alone (though it does increase experimental set-up time).

Finally, functional near-infrared spectroscopy (fNIRS) is a powerful technique in which near-infra-red light is used to probe blood oxygenation in the brain. The technique is non-invasive, wearable and functional data can be collected in multiple postures and during movement. fNIRS has limited spatial precision and cannot probe electrophysiological signals (meaning temporal resolution is limited by the latency and longevity of haemodynamic response). However, because it enables measurement of a fundamentally different metric of brain function, it complements OPM-MEG and there exists the possibility that it could be combined with OPM-MEG, to enable simultaneous electrophysiological and haemodynamic measures within a single wearable device. This may offer additional clinical benefits in pwMS.

## 5 Conclusion

We have demonstrated that a novel OPM-MEG system, with a lightweight helmet and backpack-mounted electronics, is suitable for use in pwMS. Our system enabled collection of MEG data in participants while sitting and standing. Previously established markers of MS – including a delayed beta rebound and diminished visual gamma oscillations – were measurable using OPMs. Further, we found that standing (compared to sitting) changed both functional connectivity and beta band power in the sensorimotor network, highlighting the potential of naturalistic tasks to discover novel disease biomarkers. Overall, our paper confirms that OPM-MEG is a useful means to investigate neural substrates underlying MS and paves the way for more expansive studies of movement (e.g., turning, stepping, balancing or walking). Such studies will find application not only in MS, but across a range of neurological disorders.

## CRediT authorship contribution statement

**Benjamin J. Sanders:** Writing – review & editing, Writing – original draft, Visualization, Software, Methodology, Investigation, Formal analysis, Data curation, Conceptualization. **Christopher Gilmartin:** Writing – review & editing, Methodology, Investigation, Conceptualization. **Lukas Rier:** Writing – review & editing, Software, Methodology, Investigation, Conceptualization. **Lauren Gascoyne:** Writing – review & editing, Methodology, Investigation. **Emily McCann:** Writing – review & editing, Methodology, Investigation. **Jorge Cabrera:** Writing – review & editing, Methodology, Investigation. **James Leggett:** Writing – review & editing, Methodology, Investigation. **Niall Holmes:** Writing – review & editing, Methodology, Investigation. **Ryan M. Hill:** Writing – review & editing, Methodology, Investigation, Software. **Elena Boto:** Writing – review & editing, Methodology, Investigation, Software. **Natalie Rhodes:** Writing – review & editing, Software, Methodology, Investigation. **Clarise Castleman:** Writing – review & editing, Methodology, Investigation. **Aimee Hibbert:** Writing – review & editing, Methodology, Investigation. **Daniel C. Ford:** Writing – review & editing, Methodology, Investigation. **Holly Schofield:** Writing – review & editing, Methodology, Investigation. **Cody Doyle:** Writing – review & editing, Methodology, Investigation. **James Osborne:** Writing – review & editing, Methodology, Investigation. **David Bobela:** Writing – review & editing, Methodology, Investigation. **Vishal Shah:** Writing – review & editing, Methodology, Investigation. **Karen J. Mullinger:** Writing – review & editing, Visualization, Supervision, Conceptualization. **Kathryn Radford:** Writing – review & editing, Visualization, Supervision, Funding acquisition, Conceptualization. **Matthew J. Brookes:** Writing – review & editing, Visualization, Supervision, Funding acquisition, Conceptualization. **Nikos Evangelou:** Writing – review & editing, Visualization, Supervision, Funding acquisition, Conceptualization.

## Declaration of Competing Interest

The authors declare the following financial interests/personal relationships which may be considered as potential competing interests: L.R., N.H., and R.M.H are scientific advisors for Cerca Magnetics Limited, a company that sells equipment related to brain scanning using OPM-MEG. N.H and R.M.H also hold founding equity in Cerca Magnetics Limited. E.B. and M.J.B are directors and hold founding equity in Cerca Magnetics Limited. V.S. is the founding director of QuSpin, a commercial entity selling the OPM magnetometers used in this study. CD, DB and JO are all employees of QuSpin.

## Figures and Tables

**Fig. A1:**
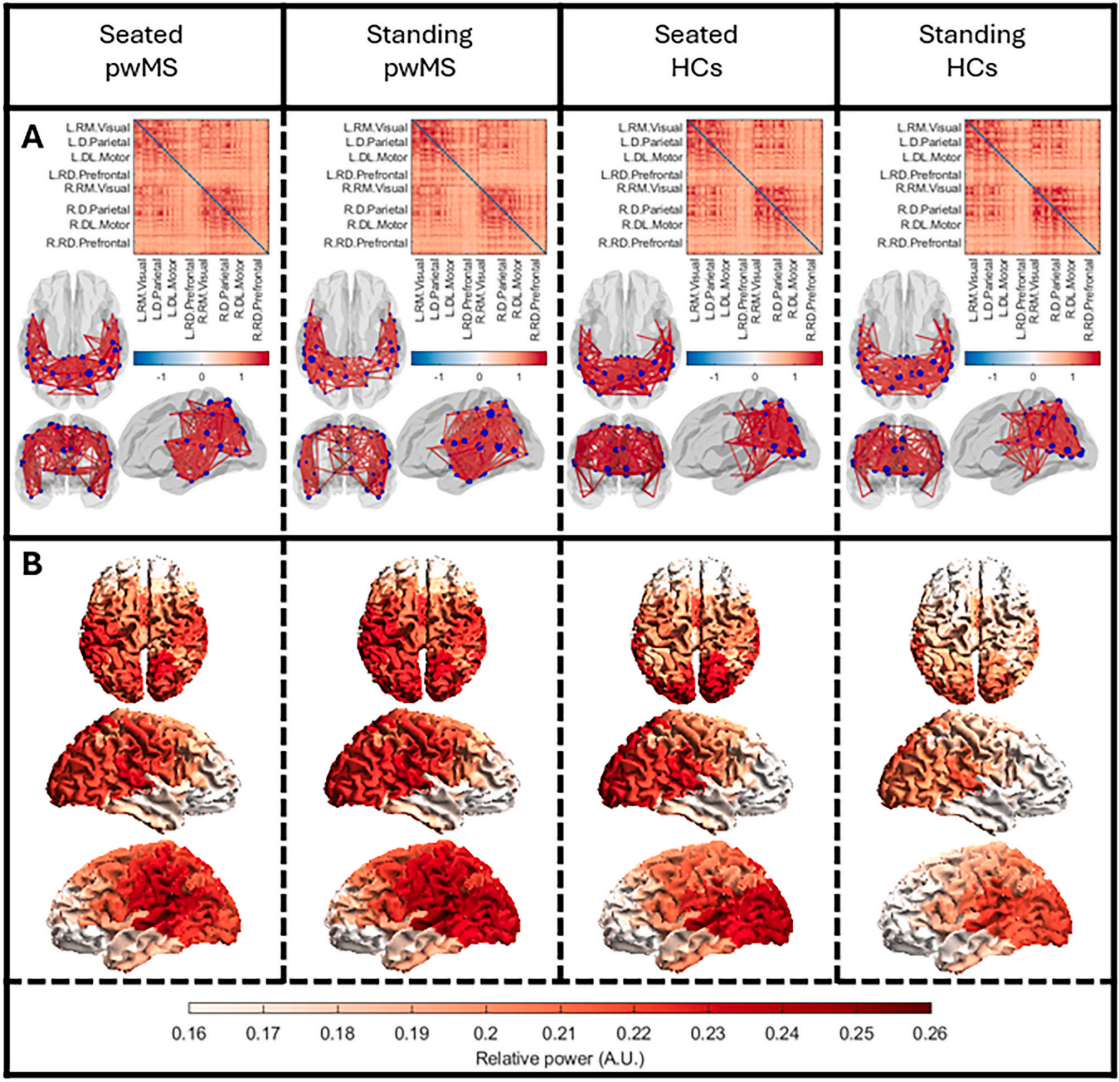
**Alpha band Resting state connectivity and relative power**. A) Connectome matrices, and corresponding glass brain plots, showing patterns of alpha band functional connectivity. B) Group averaged maps showing the spatial distribution of relative alpha power. In both A and B, the left panel shows seated pwMS, the centre left panel shows standing pwMS, the centre right panel shows seated controls, and the right panel shows standing controls.

**Fig. A2:**
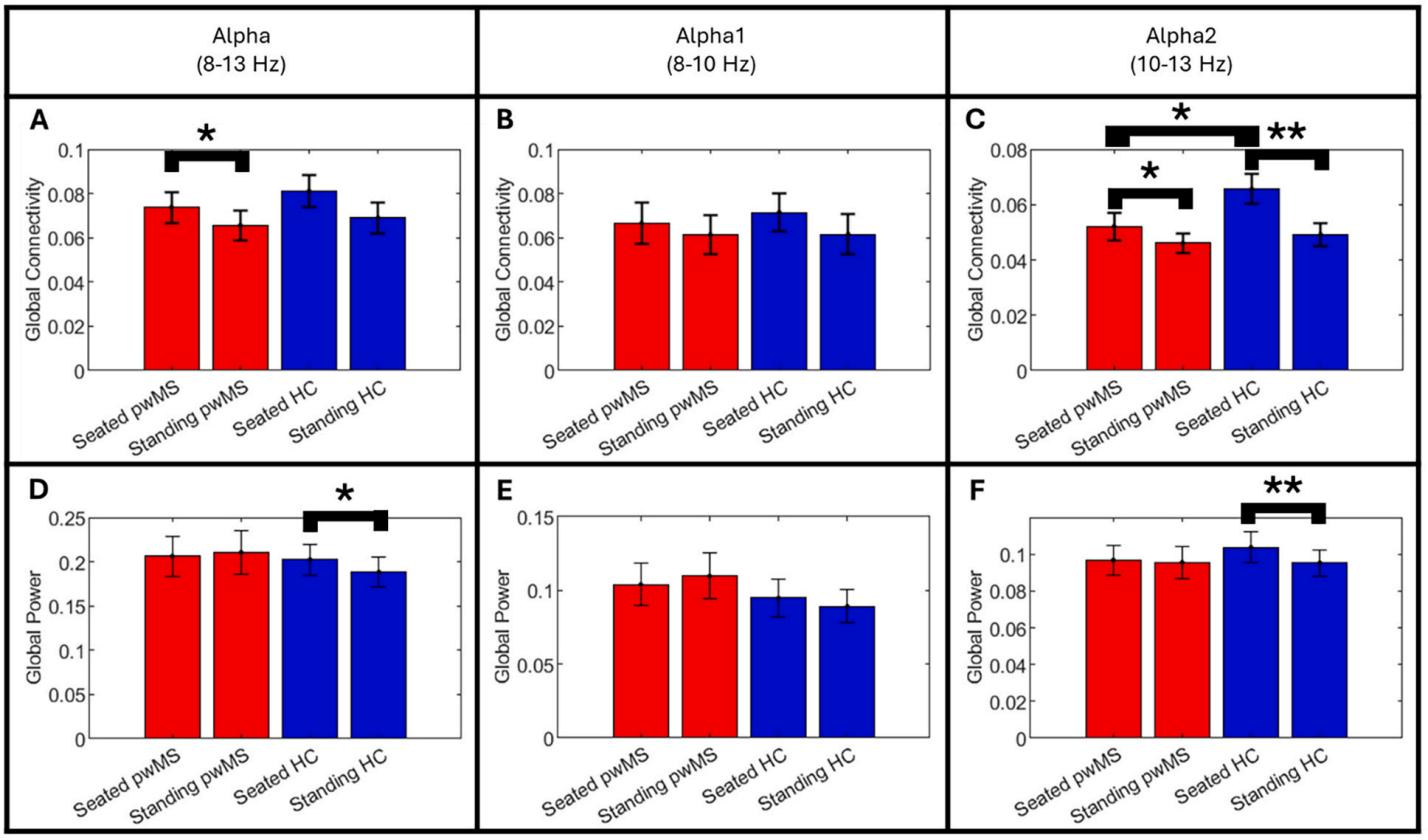
G**lobal resting state connectivity and relative power values.** A) Bar chart showing global alpha band (8–13 Hz) connectivity, for patients and controls, seated and standing. B) Same as A but for low alpha (8–10 Hz). C) Same as A but for high alpha (10–13 Hz). D) Bar chart showing global relative alpha (8–13 Hz) power, for patients and controls, seated and standing. E) Same as D but for low alpha (8–10 Hz). F) Same as D but for high alpha (10–13 Hz). For all charts, the height of the bar represents the mean of all subjects and error bars show standard error across subjects.

## Data Availability

Data and code will be made freely available upon acceptance of this manuscript, via Zenodo.

## Appendix: Resting state alpha power and connectivity

Whilst not included in our original hypotheses, there is good evidence for disruption of resting state alpha power in pwMS. Specifically, previous work has shown an increase in low (8–10 Hz) alpha power and a decrease in high (10–13 Hz) alpha power (Van Der Meer et al., 2013) in pwMS relative to controls. This cortical slowing was associated with worse cognition. Functional connectivity metrics also tended to show lower global integration in the high alpha range (Tewarie, et al., 2014). Here, we examined alpha band oscillatory power and connectivity in both seated and standing participants, hypothesising 1) slowing of the alpha frequency and diminished connectivity in pwMS and 2) a change in alpha power and connectivity when standing compared to sitting, as an explanatory analysis.

### Methods

The methodology was identical to that used in the main manuscript, with the only difference being we frequency filtered data to the low-alpha (8–10 Hz), high-alpha (10–13 Hz) and full-alpha (8–13 Hz) bands. For each band, we measured the spatial distribution of relative power as well as functional connectivity. We used a Wilcoxon sign rank test to probe differences in power and connectivity between postures, and between groups. Bonferroni correction was used to account for multiple comparisons.

### Results

Fig. A1A shows resting state alpha (8–13 Hz) connectome matrices and corresponding glass brain plots, showing the spatial signature of alpha connectivity. Fig. A1B shows the spatial distribution of resting state relative alpha power. In all cases, results are averaged across 20 participants. In both cases, the far-left panel show seated pwMS; the centre left panel shows standing pwMS; the centre right panel shows seated controls, and the far-right panel shows standing controls. Notice that, as would be expected, in all cases the alpha power and functional connectivity are maximum in the occipital regions.

In Fig. A2, panels A-C show global connectivity values (i.e., the average connectivity between all region pairs). Panels D-F show global relative power values (i.e., relative power values averaged across regions). In all six plots, mean values (across subjects) for both postures and both groups are shown, and the error bars represent standard error across participants within each group. Panels A, B and C show connectivity in the full-alpha, high-alpha and low-alpha bands respectively. Likewise, panels D, E and F show relative power in the full-alpha, high-alpha and low-alpha bands respectively.

In general, there was a trend that alpha connectivity was marginally lower when standing compared to sitting, in both groups. However, this only reached significance (following multiple comparison correction) in healthy controls in the high alpha band. High alpha band power also dropped marginally when standing, but again this only reached significance in the healthy control group.

In terms of differences between groups, there was a trend for higher seated alpha band connectivity in controls relative to pwMS, but this was not significant following multiple comparison correction. There was also a marginal increase in low alpha power in pwMS relative to controls, and this would be in agreement with existing literature, however this trend also did not reach significance.
