# Supplementary material for: OPM-MEG in multiple sclerosis: Proof of principle, and the effect of naturalistic posture
Source: Neuroimage Clin. 2025 Sep 26;48:103888. doi: 10.1016/j.nicl.2025.103888 (PMC12524172; doi:10.1016/j.nicl.2025.103888)
Supplement: Supplementary Data 1 [file mmc1.docx]

**12. SUPPLEMENTARY INFORMATION**

|  | Beta Band | Gamma Band | Resting State |
| --- | --- | --- | --- |
| Trials lost pwMS Seated | 12 ± 11 | 12 ± 9 | 7 ± 9 |
| Trials lost HCs Seated | 8 ± 6 | 10 ± 7 | 12 ± 10 |
| Channels lost pwMS Seated | 16 ± 8 | 16 ± 8 | 10 ± 7 |
| Channels lost HCs Seated | 17 ± 7 | 17 ± 7 | 15 ± 10 |
| Trials lost pwMS Standing | 10 ± 8 | 12 ± 7 | 12 ± 14 |
| Trials lost HCs Standing | 8 ± 8 | 10 ± 9 | 10 ± 12 |
| Channels lost pwMS Standing | 14 ± 7 | 14 ± 7 | 8 ± 5 |
| Channels lost HCs Standing | 16 ± 6 | 16 ± 6 | 16 ± 8 |

***Table S1:*** *Number of trials and channels designated as bad for all experiments, conditions and groups.*
